# Identification of circulating monocytes as producers of tuberculosis disease biomarker C1q

**DOI:** 10.1038/s41598-023-38889-x

**Published:** 2023-07-18

**Authors:** Paula Niewold, Douwe J. Dijkstra, Yi Cai, Delia Goletti, Fabrizio Palmieri, Krista E. van Meijgaarden, Frank A. W. Verreck, Onno W. Akkerman, Regina W. Hofland, Eveline M. Delemarre, Stefan Nierkens, Marije K. Verheul, Andrew J. Pollard, Jaap T. van Dissel, Tom H. M. Ottenhoff, Leendert A. Trouw, Simone A. Joosten

**Affiliations:** 1 Department of Infectious Diseases, Leiden University Medical Center, Leiden, the Netherlands; 2 Department of Immunology, Leiden University Medical Center, Leiden, the Netherlands; 3 Guangdong Key Laboratory of Regional Immunity and Diseases, Department of Pathogen Biology, Shenzhen University Medical School, Shenzhen, China; 4 Translational Research Unit, Department of Epidemiology and Preclinical Research, National Institute for Infectious Diseases, Rome, Italy; 5 Respiratory Infectious Diseases Unit, Clinical Department, National Institute for Infectious Diseases, Rome, Italy; 6 Section of TB Research & Immunology, Department of Parasitology, Biomedical Primate Research Centre (BPRC), Rijswijk, the Netherlands; 7 Department of Pulmonary Disease and Tuberculosis, University of Groningen, Groningen, the Netherlands; 8 Tuberculosis Center Beatrixoord, University Medical Center Groningen, University of Groningen, Groningen, the Netherlands; 9 Department of Pulmonary Diseases and Tuberculosis, University Medical Center Utrecht, Utrecht, the Netherlands; 10 Center for Translational Immunology, UMC Utrecht, Utrecht, the Netherlands; 11 Princess Máxima Center for Pediatric Oncology, Utrecht, the Netherlands; 12 Oxford Vaccine Group, Department of Pediatrics, University of Oxford and NIHR Oxford Biomedical Research Centre, Oxford, UK; 13 Centre for Infectious Disease Control, National Institute for Public Health and the Environment, Bilthoven, 3720 BA The Netherlands

**Keywords:** Vaccines, Biomarkers, Tuberculosis

## Abstract

Tuberculosis (TB) is a prevalent disease causing an estimated 1.6 million deaths and 10.6 million new cases annually. Discriminating TB disease from differential diagnoses can be complex, particularly in the field. Increased levels of complement component C1q in serum have been identified as a specific and accessible biomarker for TB disease but the source of C1q in circulation has not been identified. Here, data and samples previously collected from human cohorts, a clinical trial and a non-human primate study were used to identify cells producing C1q in circulation. Cell subset frequencies were correlated with serum C1q levels and combined with single cell RNA sequencing and flow cytometry analyses. This identified monocytes as C1q producers in circulation, with a pronounced expression of C1q in classical and intermediate monocytes and variable expression in non-classical monocytes.

## Introduction

Tuberculosis (TB) is a widespread infectious disease caused by *Mycobacterium tuberculosis* (*Mtb*). An estimated 1/3 of the global population is infected and in 2021 10.6 million new cases and 1.6 million TB-related deaths were reported^1^. Infection with *Mtb* can result in clearance, disease or infection with a lifelong risk of reactivation and development of disease^2^. Reliable and timely diagnosis of TB disease remains challenging as it is hard to distinguish from several differential diagnoses (including other respiratory diseases such as COPD, pneumonia and sarcoidosis)^3^. In addition, current gold standard immunological detection methods for *Mtb* infection, which include the Mantoux skin test and interferon gamma release assays (IGRA), cannot distinguish between past TB exposure, TB infection (TBI) and current TB disease^4^. Host gene signatures or microbiological growth assays can be used but are expensive and/or time consuming or rely on equipment not readily available in endemic areas. Therefore, easily accessible and stable biomarkers could contribute significantly to accurate and quick identification of patients that are most likely to have TB disease and initiate treatment as early as possible^5^.

C1q is a large (460-kDa) protein comprising 18 polypeptide chains (6A, 6B and 6C)^6^. C1q is known to be synthesized by cells from the monocyte lineage such as macrophages^7^, immature dendritic cells^8^ and microglia^9^, with small contributions from other cell types^10,11^. In C1q deficiencies, bone-marrow transplantation was shown in mice to be sufficient to reconstitute circulating levels of C1q^12^ and in humans hematopoietic stem cell transplantation can completely restore circulating C1q levels^13^, indicating that cells from the hematopoietic lineage are responsible for the circulating levels of C1q.

C1q has been identified as a robust biomarker for TB disease. Increased *C1QC* expression in combination with decreased *T-cell receptor-α variable gene 27* expression was identified as the gene pair most significantly able to predict TB progressors across several African cohorts^14^. C1qC protein levels were reported to be increased in a Chinese TB patient cohort^15^, although it is uncertain how C1qC levels related to the full C1q protein, as C1q is only functional if all 3 chains are present. C1q protein levels in serum are increased in individuals with TB disease and not in the most common confounder diagnoses, including other mycobacterial infections and respiratory diseases^16^. These data have been confirmed in other cohorts focusing on TB and uveitis^17^. In experimental infection models increased serum C1q coincided with classical hallmarks of progressive disease^18^. Furthermore, serum C1q has been shown to decrease upon treatment^16^. Thus, C1q is a biomarker strongly and specifically associated with TB progression and disease.

Here, we study the correlation between cell subset abundance in circulation and C1q levels in serum collected during previously performed studies of both TB-infected humans and non-human primates, to identify potential sources of C1q. Previous data have suggested monocytes as a possible source based on surface staining of peripheral blood mononuclear cells (PBMC) for C1q, which may have included C1q binding to C1q receptors on the surface^15^. Therefore, in the current study we used published single cell RNA sequencing data and performed intracellular flow cytometry to analyze C1q transcript and protein production by monocytes.

## Methods

### Ethics statement

All data from studies with human participants described here were obtained in previously performed studies and the original studies are referenced in the cohort descriptions. Human participation in this research was according to local ethical guidelines and guidelines set out by the World Medical Association’s Declaration of Helsinki. All experimental protocols were approved by an institutional and/or licensing committee, as specified below for each cohort, and methods carried out in accordance with relevant guidelines and regulations. Informed consent was obtained from all participants included in the studies.

For the Utrecht cohort, the study was reviewed and approved by Medical Research Ethics Committees-United (NL53628.100.15) and the Board of Directors of the Public Health and Diakonessenhuis, Utrecht, Netherlands. For the Leiden cohort, the study was approved by the Medical Ethics Committee of LUMC (METC project number P07.048). The Italian study was approved by the Ethical Committee of the L. Spallanzani National Institute of Infectious diseases (INMI approval numbers 02/2007 and 72/2015). The Salmonella typhi study was approved by the Indonesian National Institute of Health Research and Development (Litbangkes) and provincial authorities. The Salmonella typhi controlled infection study was approved for the primary protocol, and any study amendments, by the South-Central Oxford A research ethics committee (14/SC/1204). The study was registered with clinicaltrials.gov (NCT02192008) and was performed according to the provisions of the Declaration of Helsinki and Good Clinical Practice guidelines. The single cell RNA sequencing (scRNAseq)cohort study was approved by the Institutional Review Board of the Shenzhen University School of Medicine, China.

The non-human primate study was previously performed at BPRC which is accredited by the American Association for Accreditation of Laboratory Animal Care (AAALAC) and is compliant with European directive 2010/63/EU as well as the “Standard for Humane Care and Use of Laboratory Animals by Foreign Institutions” provided by the Department of Health and Human Services of the US National Institutes of Health (NIH, identification number A5539-01). Ethical approval for the study was obtained from the independent animal ethics committee (in Dutch: Dierexperimentencommissie, DEC), as well as BPRC’s institutional animal welfare body (in Dutch: Instantie voor Dierwelzijn, IvD).

### Patients

#### TB cohort Utrecht

80 HIV-negative adults were enrolled in this cross-sectional study from December 2015 until January 2017 through the Public Health Service of Utrecht and the Diakonessenhuis (Utrecht, Netherlands). Individuals were classified into four groups: TB disease (n = 20) with pulmonary and/ or extrapulmonary disease, TB infection without prophylactic treatment (TBI; n = 20), TBI after completion of prophylactic treatment (past TB; n = 20) and healthy controls (HCs; n = 20)^19^.

#### TB cohort Leiden

One cohort, collected by the Leiden University Medical Center (LUMC, Leiden, Netherlands), consisted of 12 TB disease patients (treated in a specialized TB clinic, Groningen, Netherlands) and 20 untreated TBI individuals who all had a positive IGRA^19^. The untreated TBI individuals within this cohort have been previously described^20^.

#### TB cohort Italy

Pulmonary TB was sputum culture-confirmed and patients were enrolled within 7 days of starting specific TB- treatment. Treated TB subjects were patients who had completed a 6-month course treatment for culture-positive pulmonary TB and who were culture-negative after 2 and 6 months of therapy. An additional group of patients was evaluated after therapy completion (1–72 months after end of therapy). TBI was defined based on positive response in Quantiferon (QFT-IT) in healthy subjects without radiological signs of active disease. TBI subjects were mainly contacts recently exposed (in the previous 6 months) to smear-positive pulmonary TB patients (15/22), however infection may have occurred also at earlier time points as no information (QFT-IT or TST) is available on any prior time points. Healthy, uninfected controls were QFT-IT negative individuals^16,21^. Cell counts were obtained on a DASIT SYSMEX Xn series.

#### Salmonella Typhi cohort

This cohort of patients has previously been described^22^, the study was performed in Indonesia between 2001–2003 and 88 patients with Salmonella Typhi were included.

#### Salmonella Typhi controlled infection

This controlled human infection study has previously been described^23^. Healthy volunteers from countries without endemic enteric fever and naïve for typhoid vaccines (Vi-polysaccharide or Ty21a) were enrolled. Participants were challenged by oral ingestion of 1–5 × 10^4^ colony forming units (CFUs) of S. Typhi Quailes strain. Blood samples were collected daily post challenge and every 12 h after diagnosis.

#### Single cell RNA sequencing cohort

Single cell RNA sequencing was performed on PBMC from 2 healthy control subjects, 2 TBI individuals and 3 patients with TB disease in China as described in^24^.

### Animals

Non-human primate (NHP) serum was available from a biobank of samples collected from earlier TB studies in healthy, purpose-bred rhesus macaques (*Macaca mulatta*) for which ethical clearance was obtained from the independent ethical authority according to Dutch law. Animals were non-vaccinated or vaccinated with BCG intradermally [BCG Danish 1331 (Statens Serum Institute, Denmark)] (n = 23), and all were experimentally infected via bronchoscopic instillation of 500 CFU of *M. tuberculosis* strain Erdman. Blood samples were collected pre-infection and at several timepoints post infection. Serum samples were previously analyzed for C1q levels^16^. Here, hematology data from week 6 and week 12 p.i. were used to study the correlation between C1q serum levels and blood cell counts. The original study was performed in accordance with the ARRIVE guidelines.

### C1q ELISA

C1q levels in serum and culture supernatants were analyzed by ELISA, as described previously^17^. Briefly, Maxisorp plates (Nunc) were coated with mouse anti-human C1q mAb 2204 (Nephrology, LUMC) in coating buffer (0.1 M NA2CO3, 0.1 M NaHCO3, pH 9.6) overnight at 4 °C. After washing plates with PBS/0.05% Tween (PBS-T, Sigma), blocking was performed with PBS/1% BSA for 1 h at room temperature. After washing, serially diluted serum was added in PBS/1% BSA/0.05% Tween (Sigma) and incubated for 1 h at 37 °C. Following incubation, plates were washed and rabbit anti-C1q (DAKO) was added and incubated for 1 h at 37 °C. Next, a goat anti-rabbit Ig HRP (DAKO) detection antibody was added and incubated for 1 h at 37 °C. After washing, the substrate reaction was performed using ABTS (Sigma) and absorbance measured at 415 nm.

### Analysis scRNAseq data

The scRNAseq dataset analyzed originates from a previously performed study^24^. Raw sequencing reads are available at the NCBI Short Read Archive (SRA) under the accession numbers SRR11038989-SRR11038995. Analysis was performed on the CellRanger files from the 2020 study, using Seurat package V4^25^. First, data of each of the individual PBMC datasets was integrated. Then possible doublets were excluded by removing the 7% of cells with the highest number of unique molecular identifiers (UMIs) and cells expressing more than 3500 genes. Cells expressing less than 500 genes or with more than 7% mitochondria-expressed genes were also excluded. Next, the data was normalized by performing a log transformation and scaled. Principle component analysis (PCA) and UMAP were performed and data visualized using feature plots to enable cluster identification.

### Flow cytometry

PBMC samples were thawed, washed with PBS and incubated with Fixable Live/Dead Blue (Invitrogen, Thermo Scientific, for 30 min at rt. Cells were then washed with PBS/0.1% BSA (Pharmacy, LUMC, the Netherlands/Sigma Aldrich, Zwijndrecht, the Netherlands) and incubated with 5% human serum in PBS for 10 min at rt. Subsequently cells were washed and incubated with antibodies against surface markers (see Table 1) in PBS/0.1% BSA for 30 min at 4 °C. For experiments comparing extracellular and intracellular C1q, anti-C1q antibody was added to the surface staining step in the samples studying extracellular C1q signal. Next, cells were washed three times and then fixed with fixation buffer A (fix/perm reagents, Nordic MuBio, ITK, Uithoorn, the Netherlands) for 15 min at rt. After fixation, cells were washed with PBS/0.1% BSA and samples assessed for intracellular C1q were incubated with anti-C1q antibody in permeabilization buffer B (Nordic MuBio) for 30 min at 4 °C. Then cells were washed three times with PBS/0.1% BSA and fixed with 1% PFA for 10 min at rt. Finally, cells were washed twice more with PBS/0.1% BSA and stored in PBS/0.1% BSA at 4 °C until acquisition. Samples were acquired on a LSR-II (Becton Dickinson, NJ, USA) at the Leiden University Medical Centre Flow Cytometry Core (FCF) and analyzed using FlowJo version 10.7 (Becton Dickinson).

**Table 1:** Overview of flow cytometry antibodies used.

Marker	Fluorophore	Clone	Concentration	Vendor
Live/dead	Fixable Blue	–	1:1000	Invitrogen
CD3	BV510	SK7	1:50	Biolegend
CD4	PerCP/Cy5.5	SK3	1:50	BD
CD8	Af700	RPA-T8	1:50	BD
CD14	PE	M5E2	1:50	BD
CD16	PE/Cy5	3G8	1:100	Biolegend
CD19	BV570	HIB19	1:50	Biolegend
CD56	PE/Cf594	NCAM16.2	1:50	BD
C1q	FITC	polyclonal	10 µL/sample	Dako

### Monocytes, M1 and M2 cells

Human peripheral blood mononuclear cells (PBMC’s) were isolated from buffycoats (Sanquin Bloodbank, the Netherlands) by ficoll density gradient centrifuge. PBMC were collected, washed with PBS, counted and incubated with CD14 microbeads (Miltenyi Biotech, the Netherlands) according to manufacturer’s protocol and isolated with an AutoMacs Pro. CD14^+^ cells were split in 3 aliquots, 1/3 of the cells were plated at 3 × 10^5^ cells per well in a 96 flatbottom plate in RPMI1640 Dutch modified supplemented with 5 mM glutamax (Gibco lifetechnologies, ThermoFisher, Bleiswijk, the Netherlands) and 10% FBS (Hyclone, UT, USA) and used as monocyte cultures; 1/3 of the CD14^+^ cells were differentiated for 6 days at 37 °C, 5% CO_2_ into M1 type macrophages by addition of 5 ng/ml GM-CSF (ThermoFisher Scientific) and the remaining cells were differentiated into M2 type cells by adding 50 ng/ml M-CSF (Miltenyi Biotec) to the culture medium. Cells were harvested on day 7 and plated in a 96 well flatbottom plate at 3 × 10^5^ cells/well.

### In vitro infection assay monocytes and macrophages

Mtb (strain H37Rv) and *M. bovis* BCG (Pasteur) were cultured in Difco Middlebrook 7H9 Broth (BD Bioscience, Erembodegem, Belgium) supplemented with 10% acid-albumin-dextrose-catalase (ADC, BD Bioscience), 0.5% Tween-80 (Sigma-Aldrich), 2% glycerol (Sigma-Aldrich). Mycobacterial cultures were diluted to an early log-phase corresponding with an OD600 of 0.25 one day prior to infection.

Monocytes, M1 and M2 cells were infected for 1 h with either live Mtb, BCG or heat killed mycobacteria (30 min, 80 °C) at 37 °C, 5% CO_2_ with a multiplicity of infection (MOI) of 10^26^. Samples were washed with PBS and incubated with RPMI 10%FBS and 30 µg/ml Gentamicin (Lonza) for 15 to 30 min to kill extracellular bacteria. Samples were washed again and incubated at 37 °C, 5% CO_2_ in medium containing 5 µg/ml Gentamycin. After 2 h the supernatant of the first samples was collected and stored at − 20 °C until further use. This process was repeated at 6, 24, 48, 96, 120 and 144 h.

### Statistics

All data were analyzed assuming a non-Gaussian distribution, and therefore nonparametric testing was applied. Sample numbers and statistical tests are described in detail in figure legends. In general, group comparisons were performed using Kruskal–Wallis test, with Dunn’s multiple comparisons correction where appropriate. Spearmans´ test was used to analyze correlations. All analyses were performed using GraphPad Prism version 9.3.1 (GraphPad Software Inc.).

## Results

**Figure 1:**
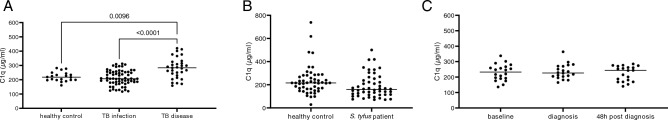
C1q serum levels are increased in patients with pulmonary tuberculosis disease but not during Salmonella Typhi infection. Serum C1q levels (µg/mL) were measured by ELISA in different cohorts; (**A**) in a cohort of individuals with TB disease (n = 31), TB infection (n = 65) and healthy controls (n = 20) from Utrecht and Leiden, (**B**) in an Indonesian cohort of Salmonella Typhi patients (n = 50) compared to endemic controls (n = 50) and (**C**) in a controlled human infection study with Salmonella Typhi*,* prior to infection (D0) (n = 20), time of diagnosis (n = 20) and 48 h after diagnosis (n = 20). Data was analyzed using a Kruskal–Wallis test.

Serum C1q has been identified as a biomarker for TB disease^14–16^. Here, we confirm in a separate cohort, that serum levels of C1q differ significantly between individuals with TB disease and those with TB infection (TBI) or in healthy uninfected controls (Fig. 1A). Previous work has shown that this elevation of serum C1q levels is not seen in patients with other respiratory disease (pneumonia), granulomatous disease (sarcoidosis) or those exposed to other mycobacteria (*Mycobacterium leprae* or Bacille Calmette Guerin)^16^. Here, Salmonella Typhi patients were also investigated, as an unrelated infection with intracellular bacteria. In Indonesian patients with Salmonella Typhi infection C1q serum levels were not elevated compared with endemic healthy controls (Fig. 1B) or during a controlled human experimental infection performed in healthy volunteers in the UK (Fig. 1C). This further extends our previous observations and suggests that the increase is specific to TB disease.

**Figure 2:**
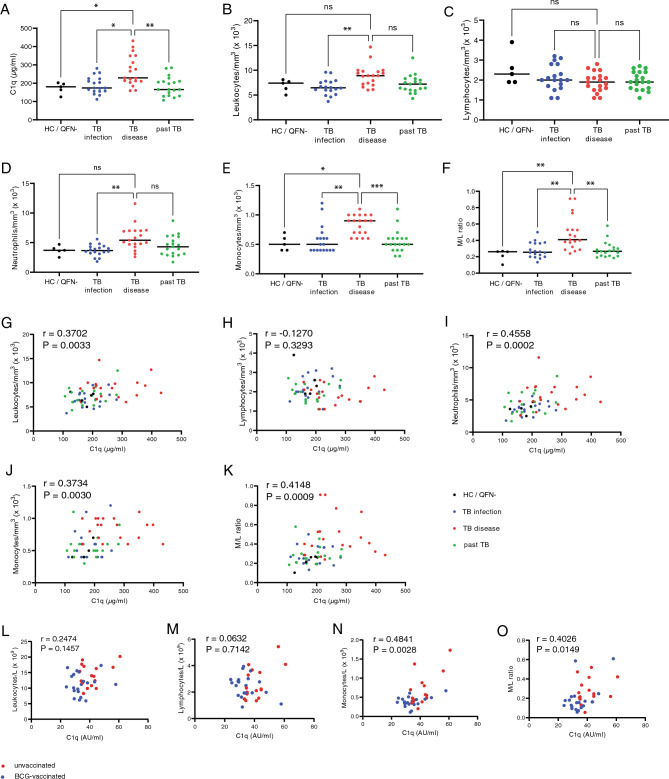
C1q serum levels correlate with circulating monocyte numbers in TB-infected humans and non-human primates. (**A**) Serum levels of C1q (µg/mL) as measured by ELISA in an Italian cohort of healthy controls (n = 5) and individuals with TB infection (n = 18), TB disease (n = 19) or past TB disease (n = 19). (**B**–**F**) Enumeration of cell populations in the circulation: leukocytes (**B**), lymphocytes (**C**), neutrophils (**D**), monocytes (**E**) and the monocyte/lymphocyte ratio (**F**). (**G**–**K**) Correlation between serum C1q (µg/mL) and cell subsets: leukocytes (**G**), lymphocytes (**H**), neutrophils (**I**), monocytes (**J**) and M/L ratio (**K**). Dots are colour-coded for the status of the individual: healthy (black), TB infection (blue), TB disease (red) or past TB (green). (**L**–**O**) Correlations between serum C1q levels and cell counts of rhesus macaques with (blue) or without (red) BCG vaccination at 6 and 12 weeks post infection with *Mtb*. Data in panel (**A**–**F**) were analyzed using Kruskal–Wallis test with a Dunn’s multiple comparisons correction and data in panel G-O were analyzed by Spearman’s correlation.

Classically, complement production is related to the liver, however, C1q in particular has previously been described to be produced mainly by circulating cellular subsets^27,28^. Here we aim to identify the origin of C1q in circulation during TB disease. Serum C1q was previously shown to be higher in individuals with TB disease, compared to healthy controls, TB infected individuals and those with past TB disease (Fig. 2A)^16^. To identify a potential source of the increased C1q levels in TB diseased individuals, cell counts in circulation from this cohort were used. This analysis revealed that leukocytes (Fig. 2B) and neutrophils (Fig. 2D), but not lymphocytes (Fig. 2C) were increased in TB disease, compared with TBI individuals. Monocyte numbers (Fig. 2E) and the monocyte/lymphocyte (M/L) ratio (Fig. 2F) were significantly increased in TB disease patients as compared with TBI individuals, as well as healthy controls and past TB patients. Next, the correlation between serum C1q levels and cell numbers in circulation was analyzed and showed a positive correlation with leukocytes (Fig. 2G), neutrophils (Fig. 2I), monocytes (Fig. 2J), M/L ratio (Fig. 2K), but not with lymphocyte numbers (Fig. 2H).

Non-human primates (NHP) are an important and physiologically relevant model to study TB disease as well as therapeutic and vaccine related interventions. In experimental infections of non-human primates, elevated levels of serum C1q were found as early as 3 weeks post infection and predicted progressive disease, prior to the formation of detectable lung lesions^18^. It is important to know whether the correlation between serum C1q levels and abundance of specific immune subsets in circulation is also represented in this model. In TB-infected rhesus macaques, serum C1q levels were positively correlated with monocyte numbers (Fig. 2N) and the M/L ratio (Fig. 2O), but not with leukocyte (Fig. 2L) or lymphocyte numbers (Fig. 2M).

**Figure 3:**
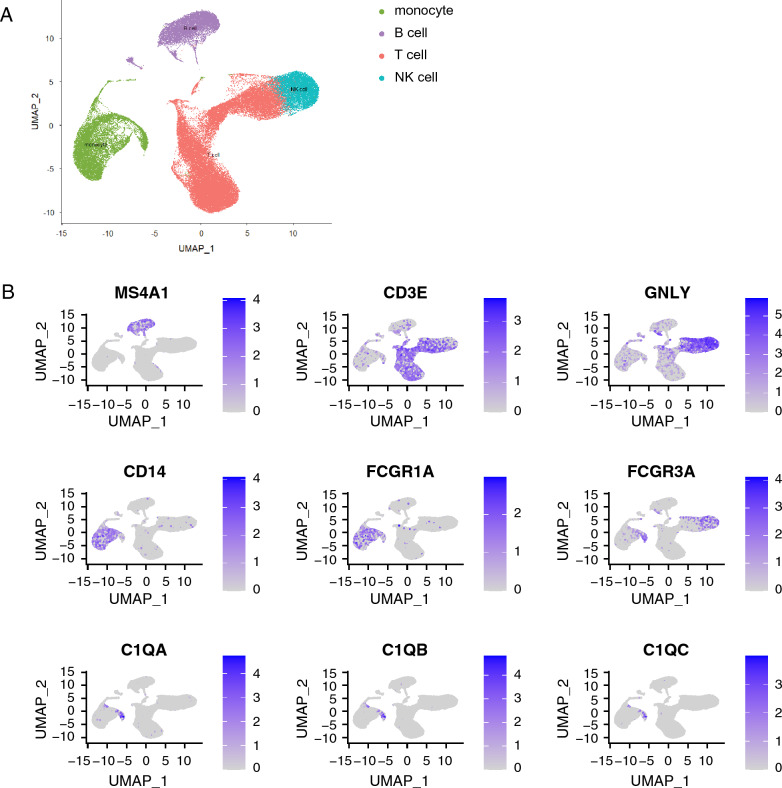
Monocytes are a main source of C1Q transcripts in single cell transcriptomic analysis. Single cell RNA sequencing data from healthy controls (n = 2), TB infected (n = 2) and TB diseased individuals (n = 3) was integrated for analysis. (**A**) Following quality control steps and scaling, PCA and UMAP were performed and cell clusters shown in a dimension plot and identified as B cells (purple), T cells (red), NK cells (blue) and monocytes (green) based on their transcript expression patterns. (**B**) Expression levels of MS4A1 (encoding CD20), CD3E, GNLY, CD14, FCGR1A, FCGR3A, C1QA, C1QB and C1QC across these clusters are shown.

The consistent correlation between serum C1q levels and monocyte numbers as well as M/L ratio in both humans and non-human primates suggests monocytes are a potential contributor of the increased C1q levels in serum, but does not show causality. Therefore, C1q transcript distribution across cell subsets was interrogated in a previously published single cell RNA sequencing dataset of PBMC of healthy, TB infected and TB diseased individuals^24^. Clustering was performed and populations of B cells, T cells, NK cells and monocytes were identified based on their expression of *MS4A1* (CD20), *CD3E*, *GLNY* and *CD14* & *FCGR1A* (CD64), respectively (Fig. 3A). The expression of *C1QA*, *C1QB* and *C1QC* transcripts were visualized across these populations and showed that C1Q transcripts were expressed mainly by monocytes (Fig. 3B). Distribution of expression of the C1Q transcripts across monocyte subsets is also shown (Suppl. Fig. 1).

**Figure 4:**
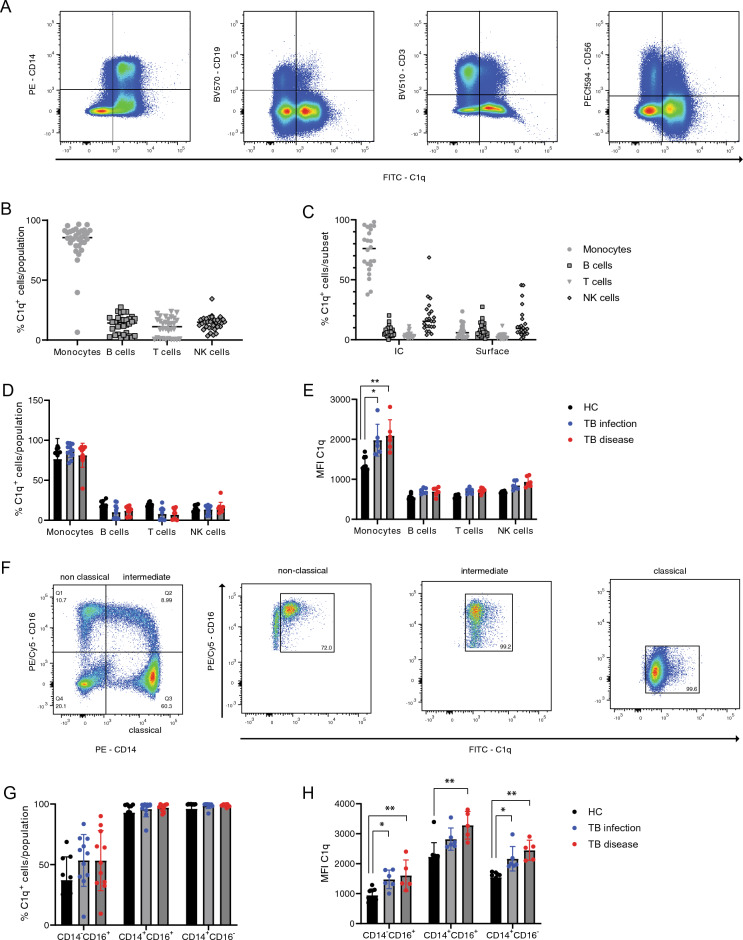
Monocytes are the main C1q-producing cells in circulation. Expression of C1q was determined by flow cytometry in healthy controls (n = 10), TB infected- (n = 11) and TB diseased individuals (n = 11) from the Leiden cohort. (**A**) Representative plots of anti-C1q staining of CD14^+^ monocytes, CD19^+^ B cells, CD3^+^ T cells and CD56^+^ NK cells. (**B**) Percentage of C1q^+^ cells per population and (**C**) as detected via intracellular (left) and surface (right) anti-C1q staining. (**D**,**E**) Quantification of percentage C1q^+^ cells (**D**) and MFI (**E**) per cell type in the different patient groups. (**F**) Representative plots of monocyte classification into CD14^−^CD16^+^ non-classical, CD14^+^CD16^+^ intermediate monocytes and CD14^+^CD16^−^ classical monocytes and their expression of C1q. (**G**,**H**) Quantification of percentage (**G**) and MFI (**H**) of C1q expression per monocyte subset in healthy controls (black), individuals with TB infection (blue) and TB disease (red). Data in panel (**D**,**E**,**G**,**H**) were analyzed using Kruskal–Wallis test with a Dunn’s multiple comparisons correction.

To determine whether protein expression of C1q was also primarily confined to monocytes, intracellular flow cytometry was performed on PBMC from healthy controls, TBI individuals and TB disease patients from the cohort shown in Fig. 1A. This showed that C1q was predominantly expressed in monocytes (Fig. 4A), with a small proportion of B cells, T cells and NK cells also staining positive for intracellular C1q (Fig. 4B). The full gating strategy is shown in Supplementary Fig. 2. As in particular monocytes also express complement receptors on the cell surface and we were interested in production in the circulation, we compared intracellular and cell-bound C1q staining by adding anti-C1q antibody to permeabilized or non-permeabilized cells from the same donor. This demonstrated that the detected C1q was largely present intracellularly and not bound to the surface, suggesting active production by monocytes rather than binding to complement receptors (Fig. 4C). To determine whether C1q production differed between patient groups, C1q positivity and MFI of intracellularly stained samples was split per patient group (Fig. 4D,E). This showed that while the percentage of C1q^+^ cells are similar for each population across patient groups, the MFI of C1q in monocytes was significantly lower in healthy controls. To determine whether a specific monocyte subset produced C1q, the monocyte population was subdivided into classical, intermediate and non-classical subsets based on marker expression, (Fig. 4F). We showed that all monocyte subsets produced C1q with non-classical monocytes as the lowest producers in both percentage of positive cells and mean fluorescence intensity (MFI), while intermediate monocytes produced the highest levels of C1q (Fig. 4G,H). These data also show TBI individuals and TB disease patients have a higher number of C1q^+^ monocytes (Fig. 4E) and level of C1q^+^ production (Fig. 4F) compared with healthy controls. Overall, we identify a positive correlation between serum C1q levels and monocyte numbers in both human and NHP with TB disease, and demonstrate production of C1q transcripts and protein by monocytes.

## Discussion

C1q is a biomarker for TB disease that can easily be detected in serum, is specific for TB disease and is not induced by BCG vaccination. Levels of C1q increase in TBI individuals progressing to TB disease, while decreasing in patients receiving treatment^14,16^. Here, we show in both human and non-human primate subjects that serum C1q levels positively correlate with monocyte numbers and M/L ratio^29^. Further experiments showed expression of C1q RNA transcripts and intracellular protein were largely confined to monocytes. While we cannot state that the increase in serum C1q levels in TB patients was solely derived from monocytes, it seems that the additive effect of increased numbers of total monocytes, increased proportion of C1q^+^ monocytes, and the higher C1q production per monocyte in TB patients all contributed to increased C1q seen compared to infected asymptomatic or uninfected healthy individuals.

C1q serum levels correlated with TB disease status and we show here that C1q is produced by cells in the circulation during TB. Intracellular staining revealed monocytes are the main producers of C1q in PBMC and a higher percentage of monocytes are C1q^+^ in TB exposed individuals. The MFI of C1q in monocytes is higher in TB infected and TB diseased individuals than in healthy controls, suggesting increased C1q production following *Mtb* exposure. This in the case for the total monocyte population, but also for each monocyte subset. Overall, C1q levels are highest in intermediate monocytes, which is in line with a recent scRNAseq study performed in different monocyte subsets in TB infected individuals pre- and post-treatment showing all C1Q genes were highly expressed in a module associated with intermediate monocyte identity^30^. The scRNAseq dataset shown here also identifies monocytes as the main C1Q transcript producing subset in PBMC. In this case non-classical monocytes showed the highest expression of C1Q genes, this discrepancy may be a result of the low number of subjects and its impact on monocyte subset deconvolution. No difference in C1q level per cell was seen between TB infected and TB disease individuals in our flow cytometry data, while serum C1q levels do differ between these patient groups. This may be explained by the higher numbers of monocytes found in TB disease individuals compared to TB infected individuals, as is shown in Fig. 2 and documented in the literature^29^. In addition, the altered activation status of monocytes in individuals with TB disease may impact the rate of C1q secretion^31^ and result in increased serum C1q. Other local or systemic sources besides monocytes may also contribute to the increased serum C1q detected in TB infected individuals.

Interestingly, in vitro infection of monocytes or macrophages did not result in C1q production (Supplementary Fig. 3), suggesting that upstream signaling leading to C1q production by monocytes in circulation may be derived from the infected lung and therefore cannot be replicated in an isolated in vitro infection of peripheral cells. This is supported by the detection of *C1QC* RNA transcripts in PBMC of TB infected individuals and lungs of *Mtb*-infected mice, while *Mtb*-infected THP cells show no *C1QC*^32^. The correlation between peripherally detectable C1q, in both serum and circulating monocytes, and the status of infection in the lung confirms it is a suitable (gene and protein) biomarker for monitoring of TB status.

In the human cohort analyzed in Fig. 2, neutrophil numbers also showed a positive correlation with serum C1q levels, but neutrophil numbers were not increased in TB patients compared to the other groups. This suggests neutrophils may be another possible source of serum C1q, although macrophages and dendritic cells are known as the main producers of C1q^28^, but this could not be assessed as we did not have access to whole blood of TB patients. Since neutrophils and monocytes have a common precursor, it may not be surprising that increasing neutrophil numbers correlate with an increase in serum C1q, even if neutrophils themselves are not contributing directly to C1q production.

The role of increased C1q in TB disease is not clear. Increased C1q may suggest a more active classical complement pathway, but the endogenous inhibitor of the classical pathway, C1-INH, was also upregulated in TB possibly counteracting increased C1q levels regarding the activity of the classical pathway^33^. Alternatively, *Mtb* may stimulate C1q production as an immune escape mechanism via one of its non-canonical functions, namely the inhibition of CD8^+^ T cells^34^. Whether soluble, secreted C1q or monocyte-associated C1q would be relevant in this inhibitory effect on CD8^+^ T cells remains to be established.

In summary, this study shows a strong positive correlation between elevated serum C1q levels in TB patients with both monocyte numbers in circulation and the M/L ratio, suggesting monocytes as a potential C1q source. This was also the case in the non-human primate model of TB infection. Further analysis showed preferential expression of *C1Q* transcripts as well as C1q protein in monocyte populations. Thus, this study confirms monocytes as a source of C1q found in the serum of TB patients and analysis of C1q levels in serum or monocytes could be considered as a potential additional parameter to monitor TB status.

## Supplementary Information

Supplementary Figures.

## Data Availability

The single cell RNA sequencing is available from the NCBI Short Read Archive (SRA) under the accession numbers SRR11038989-SRR11038995. The flow cytometry data will be made available upon reasonable request to the corresponding author. The clinical cohorts have all previously been described, we have referenced the original publications describing the cohorts.
