# Supplementary material for: Identification of circulating monocytes as producers of tuberculosis disease biomarker C1q
Source: Sci Rep. 2023 Jul 18;13:11617. doi: 10.1038/s41598-023-38889-x (PMC10354225; doi:10.1038/s41598-023-38889-x)
Supplement: Supplementary file 1 — Supplementary Figures. [file 41598_2023_38889_MOESM1_ESM.pdf]

Supplementary figure 1

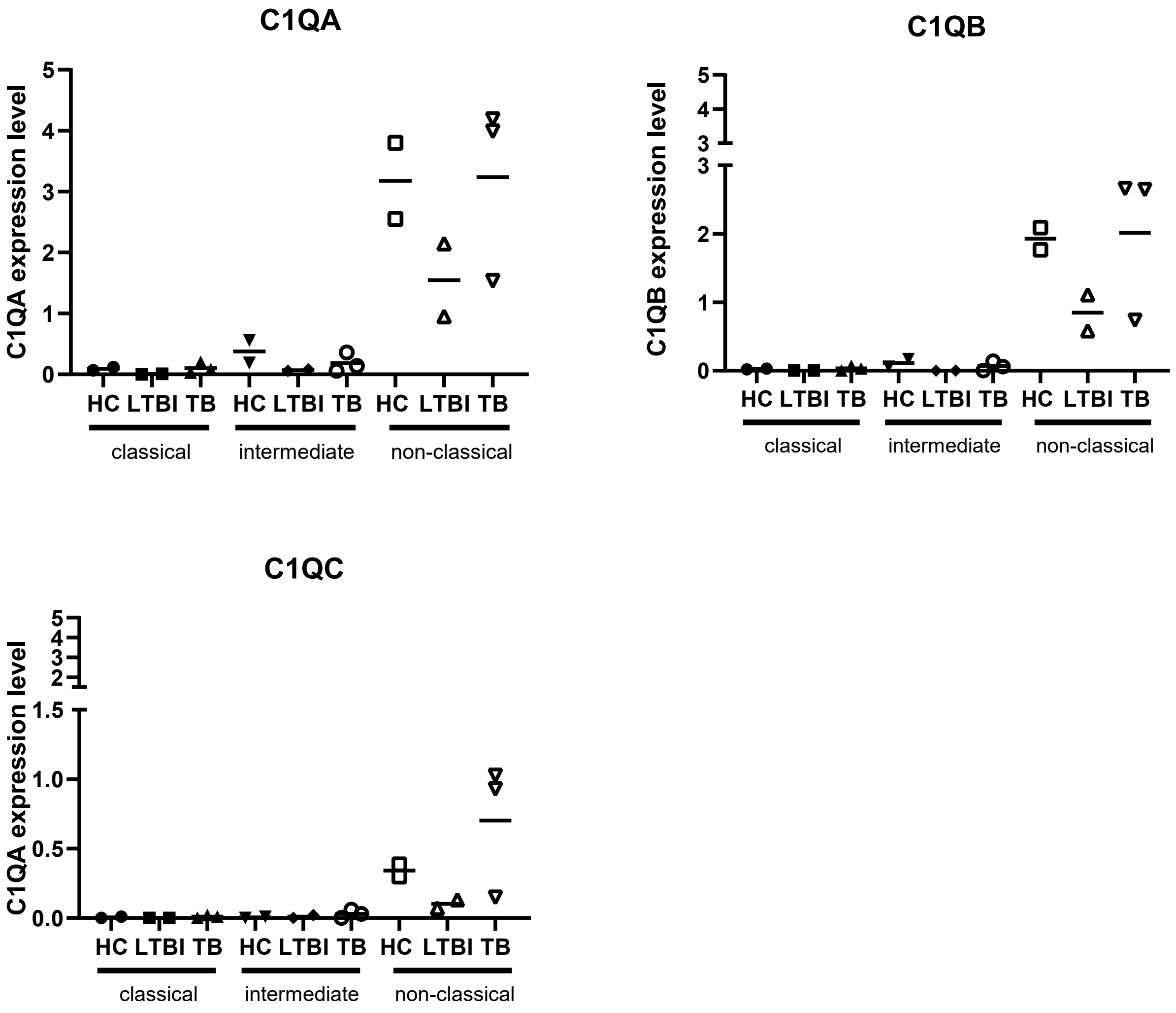

Supplementary figure 1. Single cell RNA sequencing data on C1Q transcript expression per monocyte subset and patient group. Normalized expression of C1QA, C1QB and C1QC in CD14+CD16- classical monocytes, CD14+CD16+ intermediate monocytes and CD14-CD16+ non-classical monocytes.

Supplementary figure 2

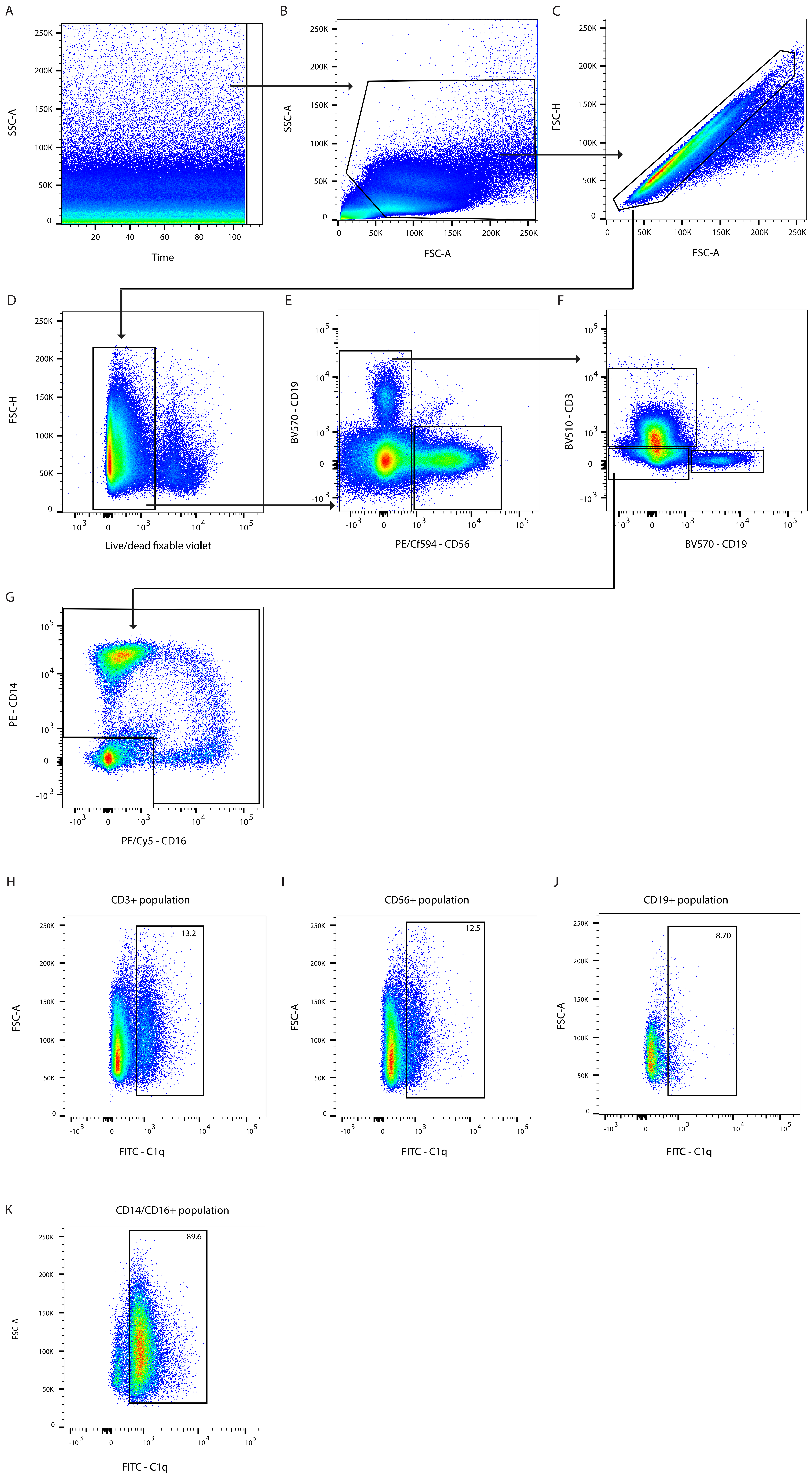

Supplementary figure 2. Gating strategy for C1q expression by PBMC. Sequential gating steps identifiied properly collected (A), whole cell (B), singlets (C). Live cells (D) were gated as CD56+ NK cells (E), CD3+ T cells (F), CD19+ B cells (F) and CD14/CD16+ monocytes (G). C1q+ cells were gated within each population(H-K).

Supplementary figure 3

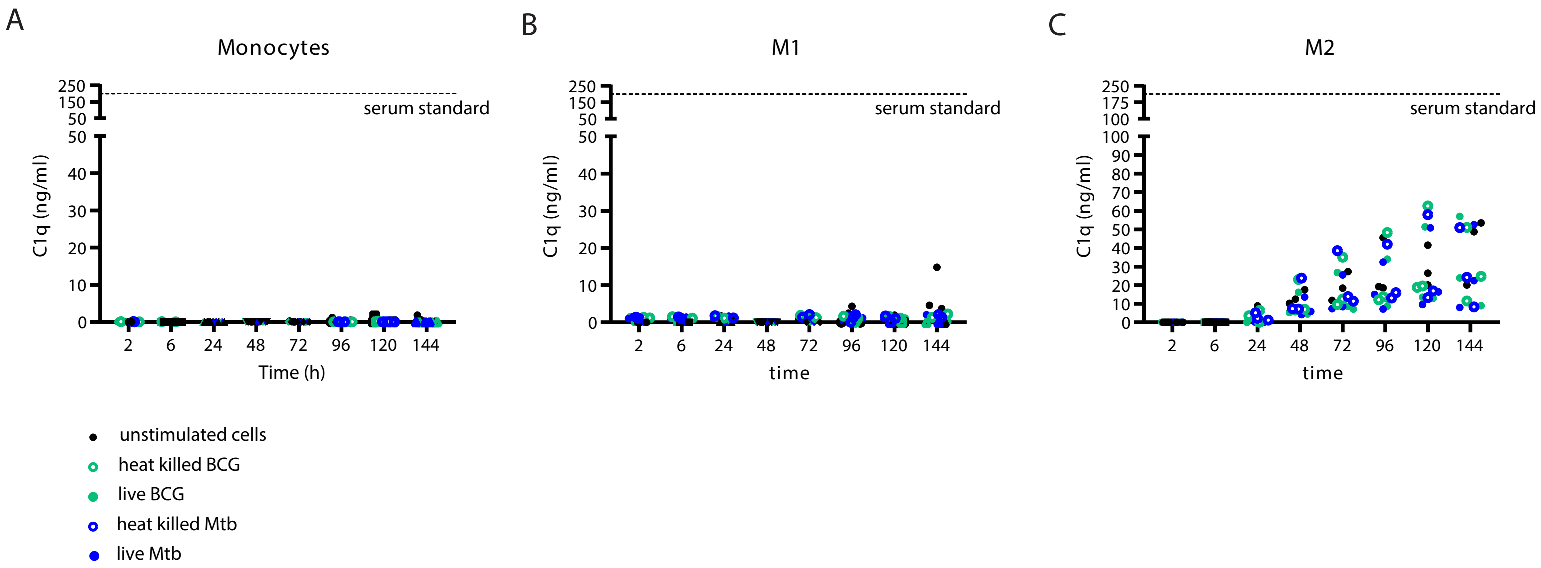

Supplementary figure 3. In vitro BCG- and Mtb-infected monocytes and macrophages do not secrete C1q. Monocytes were isolated from 3 healthy blood bank donors and samples were partly differentiated to M1 and M2 macrophages. Monocytes, M1 and M2 macrophages were then infected with live or heat-killed BCG or Mtb at an MOI of 10 for 1 hour. After killing of extracellular bacteria with gentamycin, supernatant was collected at t=2, 6, 24, 48, 72, 96, 120 and 144 hours post treatment. C1q levels in the supernatant were determined by ELISA and shown for monocytes (A), M1 macrophages (B) and M2 macrophages (C) that were unstimulated (black) or infected with live (closed circles) or heat-killed (open circles) BCG (green) or Mtb (blue). A serum standard sample was included as control (dotted line).
